# Scarlatina Treated with Oil of Turpentine

**Published:** 1856-03

**Authors:** 


*Scarlatina Treated with Oil of Turpentine.*

In a recent letter from Dr. J. S. Collings, of Boxley, Ind., he says:

"I have recently had many cases of Scarlet Fever. In treating them I used internally spts. turpentine, one part spts. nit. dulc., two parts, of which I give 15 or 20 drops to a child two years old, while the fever remains high, repeating it every two hours. I also apply externally to the throat, an equal mixture of vol. liniment and oil of turpentine, until the surface is nearly blistered. If ulceration of the throat takes place, I apply diluted spts. turpentine internally. It (the turpentine) is the best remedy I have ever found for Scarlet Fever. If the pulse was very quick and the fever high, by giving it freely for twelve hours, the turpentine would reduce both very promptly. If it will only succeed in other's hands as it has in mine, Scarlet Fever will be no more difficult to manage than the chills are with quinine. Please give it a trial.

Yours, &c."

